# Social Presence and Social Facilitation in Gambling: Some Comments on Yokomitsu et al. (2022)

**DOI:** 10.1007/s10899-022-10168-w

**Published:** 2022-11-17

**Authors:** Mark D. Griffiths

**Affiliations:** International Gaming Research Unit, Psychology Department, School of Social Sciences, Nottingham Trent University, 50 Shakespeare Street, Nottingham, NG1 4FQ UK

**Keywords:** Gambling, Gambling intensity, Gambling inhibition, Social facilitation, Social presence

## Abstract

This brief commentary adds to the recent study by paper by Yokomitsu, Kono and Takada (2022). Their study examined social presence in gambling by experimentally investigating the effects of the presence of other people on risky betting among high-risk gamblers. This commentary argues that the paper by Yokomitsu et al. provided a highly selective review on available studies and omitted many of the key studies in the area of social presence and social facilitation in which their findings could have been compared. The commentary also briefly outlines a number of studies that have I co-authored in this area over the past three decades using a variety of different methodologies (e.g., non-participant observation studies, experiments, data mining of account-based tracking data), none of which were mentioned by Yokomitsu et al. despite their clear relevance to this area.

## Introduction

The recent paper by Yokomitsuet al. (2022) examined the effects of the presence of other people on risky betting among high-risk gamblers in a laboratory experiment. In essence the study examined social presence and social facilitation in gambling (i.e., the idea that an individual’s behavior changes–and often for the better–in the presence of others). Yokomitsu et al. found in their experimental study that the presence of others stimulated risky gambling rather than inhibiting it.

Anyone reading the paper who does not know the literature on social presence and social facilitation in gambling would be given the mistaken impression that the only previous studies in this area were the laboratory experiments by Lemoine and Roland-Lévy (2017) and Rockloff and Greer (2011) because these were the only two studies that were mentioned in the paper (excluding the passing reference to Flores-Pajot et al.’s (2021) qualitative interview study which reported that gamblers said they spent more money or time gambling when other people were present). These two experimental studies both found that the presence of others appeared to inhibit gambling (which was the opposite to what Yokomitsu et al. reported).

However, there are a number of other studies that are relevant to cite but not mentioned at all. This includes (but not limited to) (i) Hardoon and Derevensky’s (2001) study of 130 children which found that girls (aged 9–13 years) increased the mean amount of money that they gambled on a computer-simulated roulette game when they gambled in a group compared to gambling on their own (as opposed to boys where there was no difference in amount of money gambled), (ii) Rockloff and Dyer’s (2007) study of 116 adults which found that individuals increased their gambling intensity on a computer-simulated slot machine when the presence of others was implied in an experimental situation, (iii) Rockloff et al.’s (2011) study of 135 adults which found that players increased their gambling intensity (as measured by gambling speed, number of trials bet, and final monetary payout) as the size of the crowd increased when playing on a simulated slot machine game on a laptop (compared to gambling alone), and (iv) Molde et al.’s (2017) study of 136 university students which found that individuals who gambled on simulated slot machines on their own gambled faster (i.e., made more bets) than those gambling alongside others. The studies by Hardoon and Derevensky (2001), Rockloff and Dyer (2007), and Rockloff et al. (2011) appear to support (at least in part) the findings of Yokomitsu et al. whereas the study by Molde et al. do not appear to support the findings of Yokomitsu et al. The paper by Yokomitsu et al. gave the impression that their findings contradicted the previous studies in the area whereas a number of experimental studies appear to concur with their findings.

## Other Research on Social Presence and Social Facilitation in Gambling

It should also be noted that none of the studies that I have authored or co-authored over the past 30 years on social presence or social facilitation in gambling were mentioned at all in Yokomitsu et al.’s paper. My own interest concerning gambling in the presence of others initially stemmed from my PhD research (1987–1990) where I spent a lot of time engaged in non-participant observation of slot machine gamblers in British amusement arcades up and down the country. One thing I observed (and made specific reference to in my early empirical studies) was how an individual’s gambling behavior would change once they realized they knew I was watching them gambling (e.g., Griffiths, 1991, 1994). I also made the same point in a later longitudinal observation study (i.e., Griffiths, 2011).

In 2003, I co-authored a study on the environmental psychology of gambling based on over ten years of observational research (Griffiths & Parke, 2003) which included our observations on social presence and social facilitation in gambling venues (e.g., amusement arcades). We noted that bystanders’ effects on gamblers’ behavior was complex and speculated based on our observations that gamblers’ behavior might be different depending on whether the person watching was a friend (and whether the friend was a gambler or not) or a stranger. We noted three main effects of being watched by friends: (i) *increased risk-taking* (where there was a need to impress friends who gambled through “risky but exciting” play in which respect was provided due to the “fearless” element in another gambler’s play); *improved skill level* (where gamblers aimed to demonstrate their skill levels to other gambling friends around them); and *increased play duration* (where groups of friends gambling on slot machines would watch each other gambling and enjoy the “secondary high” thereby staying longer in that environment). For friends who did not gamble, our findings indicated that their presence was primarily inhibitory because non-gambling friends (i) gave negative appraisals for unnecessary risk-taking and (ii) wanted to do something else other than watching others gamble.

In 2011, I co-authored a study where we experimentally examined the role social facilitation in gambling behavior between online and offline gamblers playing simulated roulette (i.e., Cole et al., 2011). A total of 38 participants played online and offline roulette either alone or alongside another gambling participant, and the players’ chip placement and amount of money bet was recorded. We found that those who gambled alongside another gambler placed more chips and made riskier bets than those who gambled alone. Those who gambled online and in the presence of others, placed the highest number of chips per bet and made the riskiest bets. The results suggest gambling alongside others led players to stake more than when playing alone which is similar to the findings of Yokomitsu et al. but was not mentioned in their paper.

In 2018, I co-authored a study using account-based tracking data to make inferences about social facilitation in gambling (i.e., Sagoe et al., 2018). In Norway, any individual who wants to gamble on products provided by *Norsk Tipping* (the government-owned the monopoly gambling operator), has to use a player card. These player cards not only track all online and offline gambling behavior on *Norsk Tipping* products (except scratch-cards), but also provides geographical data concerning the venue where the gambling took place. Using these real-world data from over 93,000 gamblers (comprising over 153,000 observations), the study examined gambling behavior in venues with different numbers of video lottery terminals (VLTs). We speculated that bigger venues with more VLTs would have larger numbers of people in the venue and/or those in the larger venues would more likely be attended by other gamblers. The findings indicated that gambling frequency was highest in venues with 2–5 VLTs (54.5%). Compared to venues with one VLT, venues with two or more VLTs were associated with gamblers placing more bets, and spending more time and money per session. However, gamblers had higher losses (albeit small) in venues with one VLT compared to venues with 2–5 VLTs. Gambling behavior appeared to reinforced more greatly in venues with multiple VLTs (compared to venues with only one terminal). We speculated that the presence of other gamblers in venues with multiple VLTs more commonly normalized gambling intensity (Rockloff et al., 2016) and that the presence of friends or other people in bigger venues prolonged the length of gambling sessions (Griffiths & Parke, 2003).

In a second study using account-based tracking data provided by *Norsk Tipping,* (i.e., Hopfgartner et al., 2021), we specifically examined the existence and strength of social facilitation among gamblers from February to May 2018. The initial dataset contained over 2.98 million sessions from over 61,000 sessions. We decided to only look at those players who engaged in at least 25 separate gambling sessions during that period (i.e., the most regular gamblers) as well as only including players who gambled at least twice when they were alone and at least twice in the presence of other gamblers. This left a dataset of 7608 gamblers comprising over 1.17 million observations. Our results showed that gamblers staked more money and played longer sessions in crowded venues. We also found that social avoiding gamblers (i.e., those who usually avoided gambling in the presence of others) gambled more when they played with their most-frequent co-gambler. Additionally, the findings indicated that social avoiding gamblers were more susceptible to social facilitation than gamblers who were familiar with crowded gambling venues.

None of these studies were cited by Yokomitsu et al. (2022) yet all have relevance to the specific topic they were investigating (i.e., gambling in the presence of others). These other omitted studies show that social presence and social facilitation have been studied using methodologies other than laboratory experiments and provide different kinds of insights into the behavior of gamblers in the presence of others.

## Data Availability

There are no data associated with this manuscript.
